# Prostatitis as initial manifestation of *Chlamydia psittaci* pneumonia diagnosed by metagenome next-generation sequencing: A case report

**DOI:** 10.1515/biol-2022-0596

**Published:** 2023-04-12

**Authors:** Mengjie Li, Biao Wang, Peng Liu, Huan Wang, Jian Zhu

**Affiliations:** Department of Respiratory and Critical Care Medicine, Cangzhou Fifth Hospital (People’s Hospital of Qingxian), Cangzhou 062650, China; Department of Thoracic Cardiovascular Surgery, General Hospital of Central Theater Command of The People’s Liberation Army, No. 627 Wuluo Road, Wuchang District, Wuhan 430070, China

**Keywords:** case report, *Chlamydia psittaci*, metagenomic next-generation sequencing, pneumonia, prostatitis, initial manifestation

## Abstract

*Chlamydia psittaci* (*C. psittaci*) pneumonia is a zoonotic infectious disease caused by *C. psittaci*, which is often underdiagnosed. The application of metagenomic next-generation sequencing (mNGS) provides an unbiased method for the detection of unknown pathogens. A 46-year-old man received empirical treatment with piperacillin-tazobactam and moxifloxacin after an initial diagnosis of prostatitis and pneumonia. However, he experienced recurrent symptoms and a cough, and a chest computed tomography (CT) showed aggravated pulmonary inflammation. Upon further questioning, the patient recalled a history of contact with pigeons, and a bronchoscope alveolar lavage fluid analysis with mNGS suggested *C. psittaci* infection. Following treatment with doxycycline, the patient’s symptoms were rapidly alleviated, and chest CT showed pulmonary lesions absorption. The patient was followed up for 1 month without any discomfort. This case highlights that initial manifestations of *C. psittaci* pneumonia may present with atypical symptoms such as prostatitis. Furthermore, mNGS can be a useful tool for the detection of rare or unknown pathogens such as *C. psittaci*.

## 1 Background

*Chlamydia psittaci* (*C. psittaci*) is a Gram-negative chlamydia that can infect a wide range of hosts, primarily transmitted via respiratory secretions and feces of birds, including parrots, pigeons, gulls, and other poultry or wild birds [1,2]. Many outbreaks of *C. psittaci* infection in humans, also known as psittacosis, have been reported worldwide [3,4,5], while sporadic cases have been reported now and then in Asia [6]. Psittacosis in humans is a nationally notifiable disease required by US Centers for Disease Control [7]. Zoonotic transmission of *C. psittaci* infection can cause various illness, ranging from subtle symptoms to severe systematic diseases, mainly affecting the lungs [8]. *C. psittaci* pneumonia is a disease resulting from *C. psittaci* infection, and the proportion of community-acquired pneumonia (CAP) due to *C. psittaci* is estimated to range from 0 to 6.7% [9]. The onset of *C. psittaci* pneumonia usually follows an incubation period of 5–14 days, which also can be several weeks, while symptomatic infections usually present with typical symptoms including fever, headache, cough, and chest distress [10].

Case reports of *C. psittaci* pneumonia have increased in recent years. However, asymptomatic infection or the absence of typical symptoms at onset may lead to undiagnosed infections, resulting in delayed or inadequate treatment. Therefore, it is essential to report and discuss cases with atypical initial manifestations that are eventually diagnosed as *C. psittaci* pneumonia to raise clinical awareness of atypical CAP and promote disease management. The metagenomic next-generation sequencing (mNGS) shows great favorability in detecting pathogens that are evasive, fastidious, and/or pose a transmission risk using conventional methods. Herein, we reported a case of *C. psittaci* pneumonia with prostatitis as the initial manifestation for the first time, which was diagnosed using mNGS.

## 2 Case presentation

**Figure 1:**
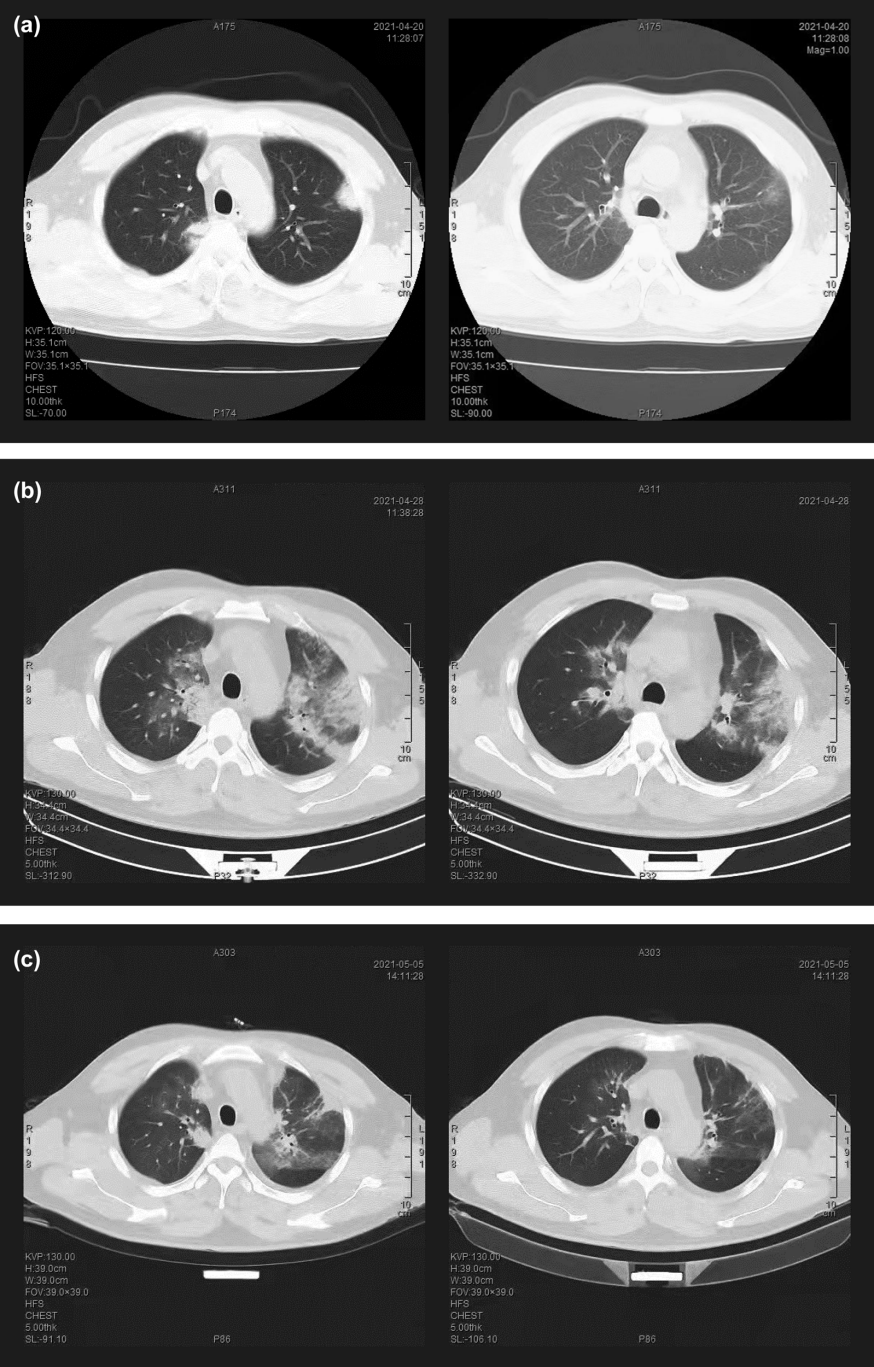
Chest CT scan during admission. (a) The chest CT images on April 20, 2021 showed nodules in the upper lobes of the lungs and a little halo sign. (b) The chest CT images on April 28, 2021 showed a range of flocculent high-density shadow increased in the lobes of bilateral pulmonary, peripheral halo sign diffused, and the lesions progressed in contrast to the images on April 20, 2021, especially the whole upper lobe of the left lung showed ground-glass opacity. (c) The chest CT images on May 5, 2021 showed that lesions are mostly absorbed and pneumonia was significantly improved.

A 46-year-old married male farmer was admitted to our hospital on April 20, 2021, due to lower urinary tract symptoms and febrile sickness. He had experienced frequent micturition and urgency, painful urination, dysuria, and discomfort of perineal swelling for about a week before admission, but did not seek medical attention. The patient reported no other symptoms such as nausea, vomiting, cough, sputum expectoration, dyspnea, fever, chills, and other discomforts. As fever occurred with a temperature of 38.5°C and lasted for 1 day without any chills and cough, he presented to the fever clinic and was referred to the urology department. The patient had no history of major diseases or infectious diseases, except for an allergy to sulfonamides. He had a smoking history of 30 cigarettes per day for 25 years and drank alcohol occasionally. On admission, his vital signs included temperature (T) of 38.7°C, pulse (P) of 92 beats/min and respiration (R) of 20 times/min, and blood pressure (Bp) of 150/90 mmHg. Pulmonary auscultation suggested that rough breath sounds in both lungs, while rhonchi and crackles were not heard. The left renal area was tender to percussion, and a digital rectal examination revealed prostate tenderness. Other physical examination results were unremarkable. Chest computed tomography (CT) scan showed double pneumonia and bilateral localized thickening (Figure 1a). Laboratory examinations of blood, urine, and prostatic fluid were performed after admission, and the results are shown in Table 1. B-Scan ultrasonography of the urinary system showed multiple small calculi in both kidneys. Considering the medical history and current examination results, prostatitis, pneumonia, and kidney calculi were tentative diagnoses. The patient was administered piperacillin-tazobactam (4.5 mg, intravenous (IV) injection, quaque 8 hours) for anti-infective treatment and symptomatic treatment for spasmolysis and relaxation of urethral smooth muscle.

**Table 1:** Laboratory test vital index

Sample	Laboratory test	Vital index
**Apr 20, 2021**		
Blood	Routine blood test	WBC 9.77 × 10^9^/L, NEUT 7.45 × 10^9^/L
	Myocardial enzymes	AST 62.97 U/L, PCT 0.1165 ng/mL
	CRP	101.86 mg/L ↑
	Blood glucose	6.56 mmol/L
Urine	Urine routine	WBC (−), WBC 0/μL, occult blood (−), RBC 0/μL
Prostate fluid	Prostate fluid test	WBC (+++)
**April 29, 2021**		
Blood	Routine blood test	WBC 8.81 × 10^9^/L, NEUT % 89.9 % ↑
	CRP	172.2 mg/L ↑
	ALB	28.8 g/L ↓
	ESR	102.10^6^ mm/h ↑
BALF	Bacterial smear test	Few amounts of Gram-positive cocci and Gram-negative bacilli
	Fungus smear test	Fungi (−)
	G test	—
	GM test	—

Abbreviations: ALB, plasma albumin; AST, aspartate aminotransferase; BALF, bronchoalveolar lavage fluid; CRP, C-reaction protein; ESR, erythrocyte sedimentation rate; GM test, galactomannan antigen test; G test, (1,3)-β-d-glucan test; NEUT %, percentage of neutrophils; NEUT, neutrophils; PCT, procalcitonin; RBC, red blood cell; WBC, white blood cell.

After initial treatment, the patient’s fever did not abate. On April 22, 2021, a consultation with the department of Respiratory and Critical Care Medicine confirmed the diagnosis of CAP and considered the possibility of mycoplasma infection. Empirical treatment with antimicrobial moxifloxacin (0.4 g, IV, quaque die) and antiviral agent oseltamivir (75 mg, peros, bis in die) was initiated, which gradually alleviated the patient’s symptoms of painful urination and dysuria, and brought his body temperature back to normal within 2 days. However, on April 28, 2021, the patient developed a fever again (T, 38.5°C) along with cough, mild chest tightness, and breathlessness. Re-examination of chest CT showed double pneumonia with localized thickening of bilateral pleura, with increased range of flocculent high-density shadow in lobes of bilateral pulmonary as compared to the initial examination (Figure 1b). After re-consultation, the patient was transferred to the department of Respiratory and Critical Care Medicine for further treatment.

**Figure 2:**
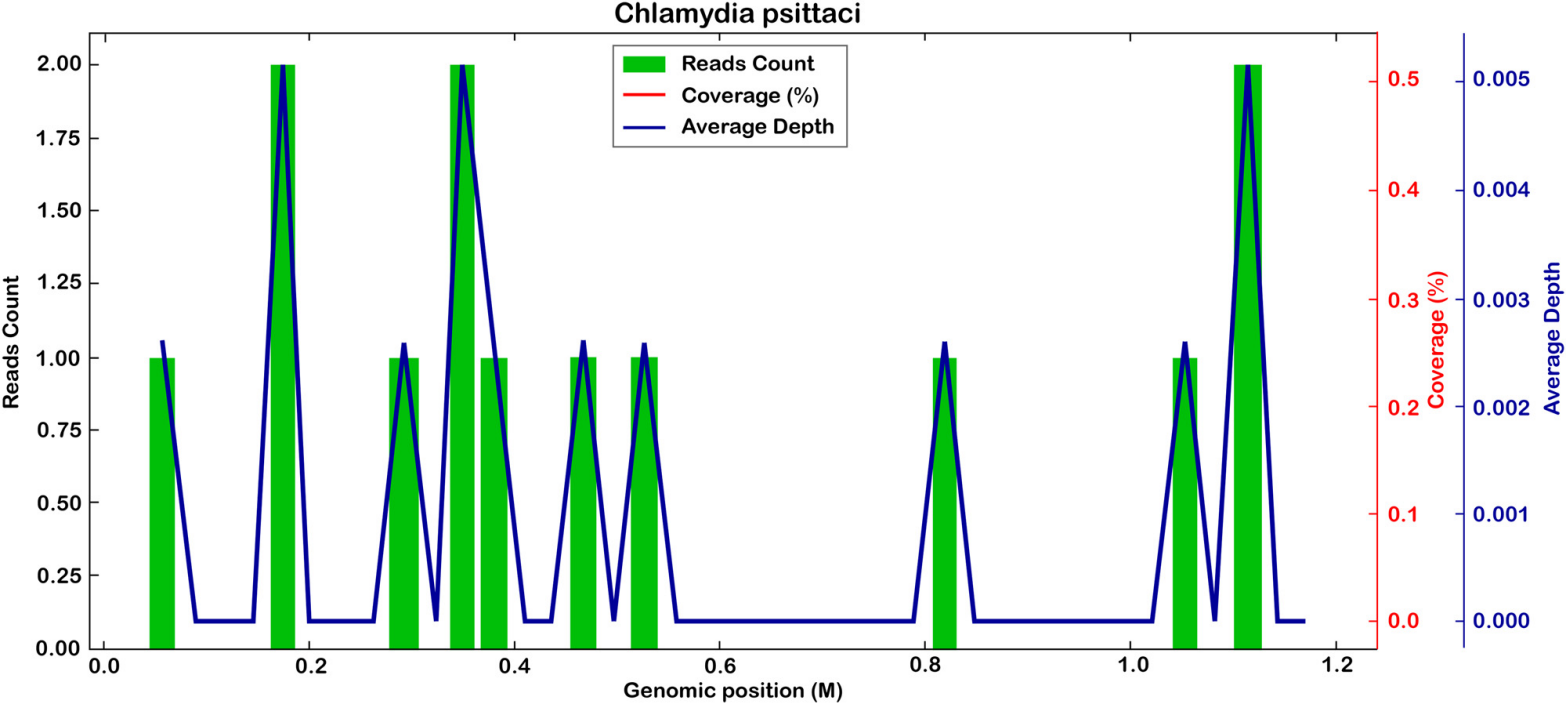
Reads count, coverage, and average depth results of *Chlamydia psittaci* suggested by metagenome next-generation sequencing in this case.

**Figure 3:**
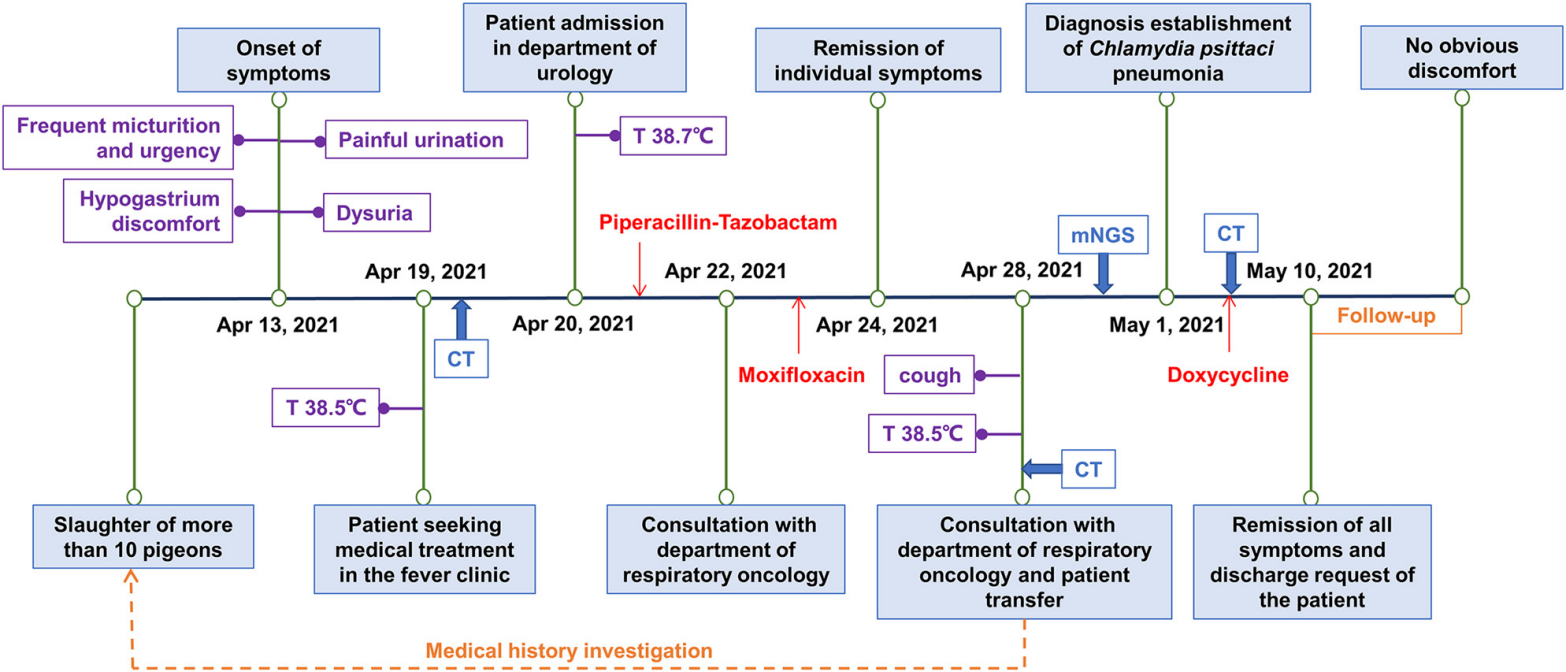
The disease progression of patient and treatment procedure. Abbreviations: CT, computed tomography; mNGS, metagenome next-generation sequencing; T, temperature.


After a detailed medical history investigation, the patient recalled having killed over 10 pigeons approximately 2 weeks before. Considering the possibility of *C. psittaci* pneumonia, electronic bronchoscopy was performed for etiological evidence, but no obvious abnormalities were noticed in the trachea and bronchus. Bronchoalveolar lavage (BAL) was performed, and bronchoalveolar lavage fluid (BALF) was collected for examination. Microbiological examination of BALF revealed a small number of Gram-positive cocci and Gram-negative bacilli. No fungi were found in BALF, while (1,3)-β-d-glucan (BDG) test (G test) and galactomannan antigen test (GM test) were negative. Routine blood examination (Table 1) showed that procalcitonin was within normal range; plasma albumin value was below the normal range, but there were no abnormalities in blood potassium, blood sodium, blood chloride, liver function, and renal function. Antibodies of mycoplasma, chlamydia, EB virus, and blood tuberculosis were all negative. Two weeks later, the chlamydia antibody was positive, and its negative may be related to the short time. BALF analysis with mNGS (Simcere Diagnostic, Nanjing, China) reported sequence reads of *Burkholderia contaminans complex* (3488), *Stenotrophomonas maltophilia* (145), *Pseudomonas aeruginosa* (22), *Candida albicans* (441), *Aspergillus flavus* (4), and *C. psittaci* (13) (Figure 2). The number of *C. psittaci* sequences detected by mNGS in the patient’s prostate fluid was 7. By joint consideration of the patient’s history of close contact with pigeons and *C. psittaci* infection suggested by mNGS, the diagnosis of *C. psittaci* pneumonia was confirmed. On May 1, 2021, doxycycline (100 mg, IV, quaque 12 hours) was administered in combination with moxifloxacin, and the fever subsided the next day. After 5 days, a re-examination of chest CT revealed that lesions were mostly absorbed and pneumonia was significantly improved (Figure 1c). After doxycycline combined with moxifloxacin treatment, the fever did not recur, respiratory symptoms subsided, and the urinary system symptoms alleviated significantly. Due to improvement, the patient was discharged on May 10, 2021. Regarding bilateral kidney calculi, he was instructed to drink plenty of water and consulted the department of urology if necessary. With a follow-up for 1 month, the patient did not have any discomfort. The disease progression of the patient and treatment procedure are shown in Figure 3.

**Informed consent:** Informed consent has been obtained from all individuals included in this study.
**Ethical approval:** The research related to human use has been complied with all the relevant national regulations, institutional policies and in accordance with the tenets of the Helsinki Declaration, and has been approved by the authors’ institutional review board or equivalent committee.

## 3 Discussion

The clinical manifestations of *C. psittaci* pneumonia vary from asymptomatic in mild cases to multiple organ failure in severe cases [11]. The atypical clinical manifestations of *C. psittaci* pneumonia may be related to the special infection mode. *C. psittaci* infection occurs through inhalation of excretion aerosol from birds or other infectious sources, which leads to the pathogen’s proliferation in reticuloendothelial cells of the liver and spleen via blood circulation, eventually affecting the lungs and possibly leading to systemic infection [8]. Previous cases have reported pneumonia with other systemic organ diseases including splenomegaly, myocarditis, keratitis, gastrointestinal symptoms, and even fatal meningitis [10,12]. To our knowledge, this is the first report of prostatitis as the initial manifestation of *C. psittaci* pneumonia. Although prostatitis is common in male patients under 50 years, its etiology is still unclear, with pathogenic factors typically including infection by a pathogen [13]. In this case, no significant abnormalities were found in routine urine tests, thus excluding urinary tract infection as a possible cause. Based on initial chest CT findings of lung inflammation and increased white blood cell count in prostatic fluid, diagnoses of pneumonia and prostatitis were made. Initially, *C. psittaci* infection was not suspected due to the lack of relevant evidence, and empirical anti-infective agents were used. However, worsening symptoms of diffuse pulmonary inflammatory lesions shown by chest CT after initial treatment suggested that the pathogen might not be common.

In the initial examination of chest CT examination, nodules were observed in the upper lobes of the lungs, distributed along the pleura, and accompanied by a slight halo sign. However, misdiagnosis as mycoplasma infection resulted in the progression of flocculent high-density shadow increased in the leaves of bilateral pulmonary and diffusion of peripheral halo. Notably, the upper lobe of the left lung showed ground-glass opacity, indicating the development of the disease. There is limited literature available on the imaging changes associated with *C. psittaci* pneumonia. While a comparative study reports ground-glass opacity as the most common imaging change [14], others have attached importance to lobar consolidation [15]. Some scholars suggest that the halo sign is a typical imaging change of this disease [16], whereas we think that the halo sign is not a specific change and can be observed in a variety of diseases such as pulmonary cryptococcosis [17]. Due to a lack of specificity in imaging, the differential diagnosis requires the identification of a variety of atypical pneumonia, including *Coxiella burnetii*, *Legionella*, *Chlamydia,* and *Mycoplasma* [18]. Despite considering the possibility of fungal infection, BALF smear, G test, and GM test showed no abnormalities.

Clinical diagnosis of *C. psittaci* pneumonia can be challenging due to the nonspecific imaging findings and patient symptoms. Traditionally, psittacosis is diagnosed based on clinical presentation, culture, and serological tests, which include complement fixation (CF), microimmunofluorescence (MIF), or enzyme(−linked) immunosorbent assay (ELISA). However, these methods can be ambiguous and deceptive due to cross-reactivity with other species, and culture is often used to confirm the results of serological tests [19]. Molecular tests, such as polymerase chain reaction (PCR), have become more popular due to their sensitivity and specificity for targeted detection [10], replacing serological methods in some cases. However, these methods require a predetermined assumption of pathogen target, which may lead to missing rare pathogens. In this case, the patient did not report a history of contact with pigeons at admission; meanwhile, we neglected the occupational characteristics of the patient as a farmer and did not pay attention to the possibility of *Chlamydia* infection, resulting in an aggravation of the patient’s condition. *C. psittaci* infection suggested by mNGS provided guidance for final diagnosis and effective treatment. mNGS can characterize all DNA or RNA existing in the samples and construct the isolated DNA and RNA libraries, so as to analyze the human host genome or transcripts in the whole microbial group and patient samples [20]. The advantage of mNGS is that it can detect any part of the genome unbiasedly to quickly identify potential pathogens, especially rare ones. The patient may not have been clearly diagnosed in this case if we did not perform mNGS. Reports of *C. psittaci* pneumonia detected by mNGS have increased gradually in recent years [12,21,22]. But it also has drawbacks. In addition to expensive and time-consuming deficiencies, not all genomes suggested by mNGS are available. Other limitations include host genomic predominance of sequence reads in patient samples (reduces sensitivity) and lower clinical utility. In the present case, counting of sequence reads *Burkholderia contaminans complex* and *Candida albicans* were much higher than that of *C. psittaci*, but *Burkholderia contaminans* had a limited effect on doxycycline and moxifloxacin, and this patient had an excellent effect on doxycycline, confirming the unlikelihood of *Burkholderia contaminans* infection. The number of *C. psittaci* sequence detected by mNGS of prostatic fluid was 7, and we finally established the diagnosis of *C. psittaci* pneumonia based on the patient’s history of pigeon exposure. Overall, mNGS is a novel technique that facilitates the diagnosis of unknown pathogens, but its data still need to be interpreted in conjunction with clinical interpretation and clinical standardization is also necessary.

During the therapy procedure, piperacillin-tazobactam was used for initial treatment, which is a typical antibiotic regimen for acute bacterial prostatitis [23]. However, since the patient’s symptoms did not improve, moxifloxacin was administered. Moxifloxacin, as a fourth-generation fluoroquinolone, has a broad spectrum of efficacy against Gram-positive bacteria and atypical pathogens, such as *Mycoplasma* and *Chlamydia* [24]. However, moxifloxacin alone is less effective in treating *C. psittaci* pneumonia, as demonstrated by the continued disease progression in this case, despite its reported effectiveness [12]. The recommended first-line treatment for *C. psittaci* pneumonia is tetracycline antimicrobials, such as doxycycline, while macrolide antimicrobials are considered an alternative for patients who are contraindicated with tetracyclines [10]. After *C. psittaci* infection was detected by mNGS and treatment with doxycycline combined with moxifloxacin, patient’s condition was obviously controlled and the imaging also showed obvious absorption of the lesions in this case. Doxycycline combined with moxifloxacin in the treatment of *C. psittaci* pneumonia has been reported in some cases [12,21], but it is not clear whether the combination is more effective than the single drug, and further research is needed.

## 4 Conclusions

*C. psittaci* pneumonia may present with atypical symptoms such as prostatitis. In cases where the initial empirical anti-infective therapy for atypical CAP is ineffective, mNGS is a promising method for identifying the pathogen and providing guidance for precise clinical treatment.

## References

[1] Hogerwerf L, Roof I, de Jong MJK, Dijkstra F, van der Hoek W. Animal sources for zoonotic transmission of psittacosis: A systematic review. BMC Infect Dis. 2020;20(1):192. 10.1186/s12879-020-4918-y PMC7057575 32131753

[2] Stokes HS, Berg ML, Bennett ATD. A review of chlamydial infections in wild birds. Pathogens. 2021;10(8):948. 10.3390/pathogens10080948 PMC8398480 34451412

[3] Laroucau K, Aaziz R, Meurice L, Servas V, Chossat I, Royer H, et al. Outbreak of psittacosis in a group of women exposed to Chlamydia psittaci-infected chickens. Eurosurveillance. 2015;20(24):21155. 10.2807/1560-7917.es2015.20.24.21155 26111240

[4] Polkinghorne A, Weston KM, Branley J. Recent history of psittacosis in Australia: Expanding our understanding of the epidemiology of this important globally distributed zoonotic disease. Intern Med J. 2020;50(2):246–9. 10.1111/imj.14726 32037712

[5] Shaw KA, Szablewski CM, Kellner S, Kornegay L, Bair P, Brennan S, et al. Psittacosis Outbreak among Workers at Chicken Slaughter Plants, Virginia and Georgia, USA, 2018. Emerg Infect Dis. 2019;25(11):2143–5. 10.3201/eid2511.190703 PMC6810211 31625859

[6] Kozuki E, Arima Y, Matsui T, Sanada Y, Ando S, Sunagawa T, et al. Human psittacosis in Japan: Notification trends and differences in infection source and age distribution by gender, 2007 to 2016. Ann Epidemiol. 2020;44(2):60–3. 10.1016/j.annepidem.2020.03.001 32253059

[7] Adams DA, Thomas KR, Jajosky RA, Foster L, Baroi G, Sharp P, et al. Summary of notifiable infectious diseases and conditions–United States, 2015. MMWR Morb Mortal Wkly Rep. 2017;65(53):1–143. 10.15585/mmwr.mm6453a1 28796757

[8] Radomski N, Einenkel R, Müller A, Knittler MR. Chlamydia-host cell interaction not only from a bird’s eye view: Some lessons from Chlamydia psittaci. FEBS Lett. 2016;590(21):3920–40. 10.1002/1873-3468.12295 27397851

[9] Hogerwerf L, De Gier B, Baan B, Van Der Hoek W. Chlamydia psittaci (psittacosis) as a cause of community-acquired pneumonia: A systematic review and meta-analysis. Epidemiol Infect. 2017;145(15):3096–105. 10.1017/S0950268817002060 PMC9148753 28946931

[10] Balsamo G, Maxted AM, Midla JW, Murphy JM, Wohrle R, Edling TM, et al. Compendium of measures to control chlamydia psittaci infection among humans (Psittacosis) and pet birds (Avian Chlamydiosis), 2017. J Avian Med Surg. 2017;31(3):262–82. 10.1647/217-265 28891690

[11] Zhang H, Zhan D, Chen D, Huang W, Yu M, Li Q, et al. Next-generation sequencing diagnosis of severe pneumonia from fulminant psittacosis with multiple organ failure: a case report and literature review. Ann Transl Med. 2020;8(6):401–6. 10.21037/atm.2020.03.17 PMC7186658 32355845

[12] Gu L, Liu W, Ru M, Lin J, Yu G, Ye J, et al. The application of metagenomic next-generation sequencing in diagnosing Chlamydia psittaci pneumonia: a report of five cases. BMC Pulm Med. 2020;20(1):65–71. 10.1186/s12890-020-1098-x PMC7077129 32178660

[13] Khan FU, Ihsan AU, Khan HU, Jana R, Wazir J, Khongorzul P, et al. Comprehensive overview of prostatitis. Biomed Pharmacother. 2017;94:1064–76. 10.1016/j.biopha.2017.08.016 28813783

[14] Itoh I, Ishida T, Hashimoto T, Arita M, Osawa M, Tachibana H, et al. Chest radiograph of atypical pneumonia: Comparison among Chlamydia pneumoniae. Pneumonia, ornithosis, and Mycoplasma pneumoniae pneumonia. Kansenshogaku Zasshi. 2000;74(11):954–60. 10.11150/kansenshogakuzasshi1970.74.954 11140079

[15] Shen L, Tian XJ, Liang RZ, Cheng Y, Kong XL, He F, et al. Clinical and imaging features of Chlamydia psittaci pneumonia: An analysis of 48 cases in China. Zhonghua Jie He He Hu Xi Za Zhi. 2021;44(10):886–91. 10.3760/cma.j.cn112147-20210127-00082 34565115

[16] Hochhegger B, Marchiori E, Irion KL, Santos de Melo G, Mendes F, Zanetti G. Psittacosis presenting as a halo sign on high-resolution computed tomography. J Thorac Imaging. 2009;24(2):136–7. 10.1097/RTI.0b013e318191987e 19465839

[17] Deng H, Zhang J, Li J, Wang D, Pan L, Xue X. Clinical features and radiological characteristics of pulmonary cryptococcosis. J Int Med Res. 2018;46(7):2687–95. 10.1177/0300060518769541 PMC6124262 29848126

[18] Cunha BA. The atypical pneumonias: Clinical diagnosis and importance. Clin Microbiol Infect. 2006;12(Suppl 3):12–24. 10.1111/j.1469-0691.2006.01393.x PMC7128183 16669925

[19] Nieuwenhuizen AA, Dijkstra F, Notermans DW, van der Hoek W. Laboratory methods for case finding in human psittacosis outbreaks: A systematic review. BMC Infect Dis. 2018;18(1):442–57. 10.1186/s12879-018-3317-0 PMC6118005 30165831

[20] Gu W, Miller S, Chiu CY. Clinical metagenomic next-generation sequencing for pathogen detection. Annu Rev Pathol. 2019;14:319–38. 10.1146/annurev-pathmechdis-012418-012751 PMC6345613 30355154

[21] Shi Y, Chen J, Shi X, Hu J, Li H, Li X, et al. A case of chlamydia psittaci caused severe pneumonia and meningitis diagnosed by metagenome next-generation sequencing and clinical analysis: A case report and literature review. BMC Infect Dis. 2021;21(1):621–8. 10.1186/s12879-021-06205-5 PMC8243071 34193063

[22] Chen X, Cao K, Wei Y, Qian Y, Liang J, Dong D, et al. Metagenomic next-generation sequencing in the diagnosis of severe pneumonias caused by Chlamydia psittaci. Infection. 2020;48(4):535–42. 10.1007/s15010-020-01429-0 PMC7223968 32314307

[23] Coker TJ, Dierfeldt DM. Acute bacterial prostatitis: Diagnosis and management. Am Fam Phys. 2016;93(2):114–20. 26926407

[24] Moxifloxacin. LiverTox: Clinical and research information on drug-induced liver injury. Bethesda (MD): National Institute of Diabetes and Digestive and Kidney Diseases; 2012. 31643176

